# Evaluating the effectiveness of a training program that builds teachers’ capability to identify and appropriately refer middle and high school students with mental health problems in Brazil: an exploratory study

**DOI:** 10.1186/1471-2458-14-210

**Published:** 2014-02-28

**Authors:** Marlene A Vieira, Ary A Gadelha, Taís S Moriyama, Rodrigo A Bressan, Isabel A Bordin

**Affiliations:** 1 Department of Psychiatry, Universidade Federal de São Paulo (UNIFESP), Rua Borges Lagoa 570, 1° andar, São Paulo, SP CEP: 04038-030, Brazil; 2 Department of Psychiatry, Laboratório Interdisciplinar de Neurociências Clínicas (LiNC), Universidade Federal de São Paulo (UNIFESP), Rua Borges Lagoa 570, 1° andar, São Paulo, SP CEP: 04038-030, Brazil; 3 National Institute of Developmental Psychiatry for Children and Adolescents-INPD-CNPq, São Paulo, Brazil

**Keywords:** Mental health, School health, Adolescence, Education, Primary and secondary, Professional training

## Abstract

**Background:**

In Brazil, like many countries, there has been a failure to identify mental health problems (MHP) in young people and refer them to appropriate care and support. The school environment provides an ideal setting to do this. Therefore, effective programs need to be developed to train teachers to identify and appropriately refer children with possible MHP. We aimed to evaluate teachers’ ability to identify and appropriately refer students with possible MHP, and the effectiveness of a psychoeducational strategy to build teachers’ capability in this area.

**Methods:**

To meet the first objective, we conducted a case-control study using a student sample. To meet the second, we employed longitudinal design with repeated measures before and after introducing the psychoeducational strategy using a teacher sample. In the case control study, the Youth Self-Report was used to investigate internalizing and externalizing problems. Before training, teachers selected 26 students who they thought were likely to have MHP. Twenty-six non-selected students acted as controls and were matched by gender, age and grade. The underlying principle was that if teachers could identify abnormal behaviors among their actual students, those with some MHP would likely be among the case group and those without among the control group. In the longitudinal study, 32 teachers were asked to evaluate six vignettes that highlighted behaviors indicating a high risk for psychosis, depression, conduct disorder, hyperactivity, mania, and normal adolescent behavior. We calculated the rates of correct answers for identifying the existence of some MHP and the need for referral before and after training; teachers were not asked to identify the individual conditions.

**Results:**

Teachers were already able to identify the most symptomatic students, who had both internalizing and externalizing problems, as possibly having MHP, but teachers had difficulty in identifying students with internalizing problems alone. At least 50.0% of teachers learned to identify hypothetical cases as problematic and to make the appropriate referral, and 60.0% of teachers who before training could not identify normal adolescence learned to do so.

**Conclusions:**

The strategy was partially effective but could be improved mainly by extending its duration, and including discussion of actual cases.

## Background

On July 13, 1990 the UN Convention on the Rights of the Child was incorporated into Brazil’s national legislation through law 8.069, which established the Child and Adolescent Statute (*Estatuto da Criança e do Adolescente*). This Statute ensured the full protection of children and adolescents, and also directed and issued measures to provide for their access to health care and education [1]. The Child and Adolescent Statute is a fundamental step in ensuring the wellbeing of children and adolescents, and this Statute has led to the more complex task of turning it into a reality.

Mental health disorders are not only a major cause of disability and suffering, but also contribute to general health problems and the associated healthcare costs [2-4]. Many mental health problems identified in adult life have begun in childhood and adolescence; therefore, it is particularly important to screen for these early problems and try to prevent progression by providing early care. Worldwide, child and adolescent mental health has increasingly become a priority, particularly in the fields of mental, neurological and substance-use disorders [3]. However, the mental health needs of children and adolescents continue to be neglected, especially in low- and middle-income countries [4].

Mental health problems in childhood and adolescence are common in Brazil and detrimental to academic performance [5]. The prevalence rates of mental health problems in this age group have been measured using screening instruments. The rate of mental health problems were found to vary according to the type of informant: parents (13.5–35.2%), adolescents (12.6–13.1%), and teachers (8.3–10.3%) [5]. Studies that combined data provided by two or more informants found higher prevalence rates (22–24.6%) [5]. When diagnostic interviews based on ICD-10 or DSM-IV criteria were used, the prevalence rates of one or more mental health disorders were 7–12.7% [5].

Brazilian government institutions admit that child and adolescent mental health is a public health issue that should be addressed and integrated into Brazil’s Unified Health System (*Sistema Único de Saúde*) as part of the development of Brazilian general mental health policy. Over the last 10 years, progress has been made with new services being established, such as child and adolescent psychosocial attention centers (*Centro de Atenção Psicossocial Infantil*), and strategies for intersectoral cooperation being developed [6]. However, the stigma that surrounds mental disorders and the lack of awareness about mental illness hampers the use of such centers [7]. The use of schools as a platform for increasing awareness, facilitating early detection and referring cases to mental health care has been highlighted as a promising alternative [8,9].

Given that education is now compulsory for all Brazilians aged 4 to 17 years, schools are where children and adolescents with emotional and behavioral problems are most likely to be identified. Hence, it is important that educators are capable of identifying those schoolchildren who may have early indicative signs of mental health problems. Referral to appropriate health services would not only reduce the disease burden, but also prevent the progression of psychopathology in the long term. Closer collaboration between the areas of health and education could benefit public school students by promoting mental health and well-being. This would facilitate access to health services, increase treatment adherence [10] and ultimately reduce school failure and dropout rates, especially in those communities where resources are scarce. However, the main problem is that this collaboration relies on teachers having the ability to identify and appropriately refer children with possible mental health problems, but no specific training programs are currently being offered to teachers. Therefore, appropriate and effective training programs need to be developed.

The first aim of this study was to evaluate teachers’ current ability to identify and appropriately refer middle and high school students with possible mental health problems using real life examples in a case control study, and in an additional study using six vignettes, five of which were related to psychopathological conditions and one which was related to normal adolescence. The second aim was to evaluate the effectiveness of a psychoeducational strategy intended to develop public school teachers’ capability of identifying those children and adolescents who needed mental health evaluation and to make the appropriate referral.

## Methods

This was an exploratory, descriptive survey that included two investigations. First, one main longitudinal study with measures obtained before and after training to evaluate the effectiveness of a mental health training strategy for public school teachers. Second, an independent case-control study to evaluate teachers’ current ability to identify possible mental health problems within their own student population.

The longitudinal study evaluated a sample of teachers before and after training with participants acting as their own controls, which made it possible to control confounding individual characteristics. From a theoretical perspective, this study assessed whether the teachers were capable of identifying and properly referring students who required mental health attention. Comparing the data collected before and after the psychoeducational intervention, allowed us to evaluate the model’s effectiveness.

For the case-control study, teachers were asked to make a list with the names of their current students they thought may need mental health evaluation because of problematic behavior in the classroom. These students were allocated to the ‘cases’ group. The control group was selected from the remaining group of students and matched for gender, age (one year younger, same age or one year older), and grade. The underlying principle was that if teachers were able to identify abnormal behaviors among their own students, then those with some mental health problems would most probably be among cases and those without mental health problems would most probably be among controls.

### Teacher and student samples

#### Teachers’ training

Thirty-two teachers from São Paulo participated in the study, they taught students from the fifth grade through to those in the last year of high school. Of the 45 teachers eligible, 32 participated in the study (71.1%). Twelve did not complete the entire training and one refused to participate. At the moment of data collection, the Brazilian educational system classified fifth through eighth school grades as middle school and ninth through eleventh grades as high school. In public schools administered by São Paulo State, teachers with a workload above 12 classes per week are required to attend weekly compulsory pedagogical work meetings. The study inclusion criteria for teachers required teaching in fifth through eleventh grades and having to attend compulsory pedagogical work meetings.

#### Case-control study

The case-control study involved 26 cases and 26 controls. The inclusion criteria for students to enter the case-control study included: being aged 11–17 years and being enrolled in a grade between fifth grade and the last year of high school. Cases were those included on the teachers’ hypothetical lists as possibly having mental health problems.

### Instruments and procedures

Data were collected in March 2009.

#### Teacher training strategy description

Three questionnaires developed by the research team were administered in this study. The first one was used for collecting data on teachers’ characteristics before training (demographics, professional training and experience, professional satisfaction and self-assessment of vocational skills). The second questionnaire included six vignettes that highlighted behaviors indicating a high risk for psychosis, depression, conduct disorder, hyperactivity, mania, and normal adolescent behavior (presented in that order). The six vignettes were developed by a scientific committee that included psychiatrists and psychologists who prioritized behaviors that could be present in the classroom and identified by teachers. The intention was not only to test teachers’ general knowledge of mental health problems, but also to test their ability to recognize any abnormal adolescent behaviors at school. This questionnaire investigated whether the adolescents described had any mental health problems and required some sort of referral (mental health services, after-school help for pedagogical support, other, or none). The third questionnaire was a self-report qualitative evaluation of the training program.

Teachers attended a four-hour training program made up of two, two-hour sessions delivered in two consecutive weeks. The questionnaire about the vignettes was applied both before and after training to evaluate the acquisition of theoretical knowledge concerning the identification of youth with possible mental health problems and the appropriate referral of problematic youth. Teachers were not required to identify specific psychopathological conditions but only to identify the need for mental health evaluation because of a possible mental health problem.

In the first week, a standardized lecture was delivered, followed by theoretical and practical training on types of mental health problems that affect adolescents and their impact on school life. Training included differences between normal behaviors expected in adolescence and abnormal behaviors that represent signs of psychopathological conditions or risk for the development of mental disorders; and the impact of mental health problems on adolescents’ cognition, thought, behavior, feelings, and social skills.

In the second week, the psychoeducational intervention started with a brief recap of the topics covered in the first meeting, with an emphasis upon possible behavioral changes, decrease in school performance, and distress resulting from mental health problems. Information about when and where to refer students, who had signs of mental health problems, was then given to the teachers. Finally, they were asked to read the vignettes presented at the first meeting and to answer the same questions for each vignette concerning identification of the existence of some mental health problems and the need for referral, again teachers were not asked to identify the individual conditions.

At the end of this last meeting, teachers were also asked a single question, about whether they had already referred to mental health services any of the students included in their hypothetical referral lists. To identify ways to improve the training strategy given, we also sought the opinions and criticisms of the training program from the participating teachers.

The material used for the training is in Portuguese and available upon request to the corresponding author at the Federal University of São Paulo.

#### Case-control study description

All students from fifth grade to the last year of high school were told that they would be randomly selected to complete a questionnaire about adolescent mental health and after both the students and their parents’ consent had been obtained this questionnaire would be administered in groups. Students selected to participate in the study were divided into mixed groups, cases and controls, and taken to specific schoolrooms where they were asked to complete the self-report questionnaire. Student participants were blind to the fact that they were classified into case and control groups, and that the teachers had already made lists to identify cases.

Cases and their respective controls completed the Youth Self-Report (YSR), a screening questionnaire for the identification of emotional and/or behavioral problems in adolescents aged 11–18 years [11,12]. We used the Brazilian version of the YSR that was developed at *Universidade Federal de São Paulo* by Bordin and colleagues in 2002 [12]. The questionnaire corresponded to the original English version developed by Achenbach in 2001 [11]. The YSR is a screening instrument that provides a behavioral profile of the adolescent on scales which examine internalizing and externalizing problems. Cut-off points (T-scores) in these scales classify adolescents in three categories: clinical cases (> 63), borderline (60–63), and normal (< 60) [11,12]. Preliminary validity data on the YSR showed that it was capable of distinguishing between youth referred to mental health services and youth from the general population in Brazil [13].

In this study, we considered as YSR positive those students with scores in the clinical or borderline range (T score ≥ 60). According to internalizing and externalizing scale scores, they were further classified as having: internalizing problems only (anxiety and/or depression symptoms); externalizing problems only (aggressive behavior and/or rule breaking behavior); both types of problems; or no problems.

### Statistical analysis

Teachers’ data were examined in a descriptive analysis. Answers to the questionnaires given before and after the intervention were compared. Data of participating students (cases and controls) were also examined through descriptive analysis. Differences between cases and controls were identified using the chi-square statistic.

### Ethical considerations

This study was part of a larger project entitled Prodromal Schizophrenia, Depression and Bipolar Disorder in Students from São Paulo Metropolitan Area, and was approved by the Ethics Committee of *Universidade Federal de São Paulo*.

The Education Office of the São Paulo Center-South Region, responsible for the coordination of state schools in the area, authorized the development of the project. Written informed consent was obtained from all research participants to confirm their voluntary agreement to participate in the study. Written informed consent was also obtained from the participant adolescents’ parents or guardians. In addition, adolescents with YSR scores in the clinical range were referred to an outpatient clinic at the Department of Psychiatry, *Universidade Federal de São Paulo* for psychiatric evaluation.

## Results

In total 32 teachers were trained, 22 (68.8%) were female and 16 (82.8%) were older than 39 years. All of them had a university degree and taught subjects that corresponded to their area of expertise. When asked if they would change their profession if they could, 15 (46.9%) gave a negative answer. Table 1 presents demographic and professional characteristics of the participant teachers.

**Table 1:** Demographics and professional characteristics of the teachers’ sample by gender

		**Female (N = 22)**		**Male (N = 10)**		**Total (N = 32)**	
**Teachers’ sample**		**N**	**(%)**	**N**	**(%)**	**N**	**(%)**
Demographics							
	Age (years)						
	20-29	1	(4.5)	2	(20.0)	3	(9.4)
	30-39	5	(22.7)	1	(10.0)	6	(18.8)
	40-49	10	(45.5)	4	(40.0)	14	(43.8)
	50 or older	6	(27.3)	3	(30.0)	9	(28.1)
	Residing with a partner						
	Yes	17	(77.3)	5	(50.0)	22	(68.8)
	No	5	(22.7)	5	(50.0)	10	(31.3)
	Marital status						
	Married/Cohabitation	17	(77.3)	5	(50.0)	22	(68.8)
	Separated	0	(0.0)	2	(20.0)	2	(6.3)
	Widow	0	(0.0)	1	(10.0)	1	(3.1)
	Single	5	(22.7)	2	(20.0)	7	(21.90)
	Having children older than 11 years						
	Yes	9	(40.9)	5	(50.0)	14	(43.8)
	No	13	(59.1)	5	(50.0)	18	(56.3)
	Raising children older than 11 years						
	Yes	8	(36.4)	5	(50.0)	13	(40.6)
	No	14	(63.6)	5	(50.0)	19	(59.4)
	Religion						
	Catholic	16	(72.7)	8	(80.0)	24	(75.0)
	Protestant/Evangelical	1	(4.5)	1	(10.0)	2	(6.3)
	Spiritist/Kardecist	5	(22.7)	0	(0.0)	5	(15.6)
	Other	0	(0.0)	1	(10.0)	1	(3.1)
Professional characteristics							
	University degree						
	Portuguese literature	6	(27.3)	0	(0.0)	6	(18.8)
	Mathematics	5	(22.7)	3	(30.0)	8	(25.0)
	Geography	0	(0.0)	2	(20.0)	2	(6.3)
	History	2	(9.1)	1	(10.0)	3	(9.4)
	Arts	2	(9.1)	0	(0.0)	2	(6.3)
	Physical education	1	(4.5)	0	(0.0)	1	(3.1)
	Other	3	(13.6)	3	(30.0)	6	(18.8)
	Portuguese literature & other	2	(9.1)	0	(0.0)	2	(6.3)
	Geography/History & other	0	(0.0)	1	(10.0)	1	(3.1)
	Geography/Arts & other	1	(4.5)	0	(0.0)	1	(3.1)
	Subjects currently taught						
	Portuguese language	5	(22.7)	0	(0.0)	5	(15.6)
	Mathematics	5	(22.7)	3	(30.0)	8	(25.0)
	Geography	1	(4.5)	2	(20.0)	3	(9.4)
	History	2	(9.1)	1	(10.0)	3	(9.4)
	English language	1	(4.5)	0	(0.0)	1	(3.1)
	Arts	1	(4.5)	0	(0.0)	1	(3.1)
	Physical education	1	(4.5)	0	(0.0)	1	(3.1)
	Other	4	(18.2)	2	(20.0)	6	(18.8)
	Portuguese/English	2	(9.1)	0	(0.0)	2	(6.3)
	Mathematics & other	0	(0.0)	1	(10.0)	1	(3.1)
	Geography/History & other	0	(0.0)	1	(10.0)	1	(3.1)
	Experience in teaching children older than 11 years						
	Less than 4 years	0	(0.0)	0	(0.0)	0	(0.0)
	4 years or more	22	(100.0)	10	(100.0)	32	(100.0)
	Would like to change their profession*						
	Yes	15	(68.2)	2	(20.0)	17	(53.1)
	No	7	(31.8)	8	(80.0)	15	(46.9)
	Vocational skills (self-assessment)**						
	1 (lowest level skills)	0	(0.0)	0	(0.0)	0	(0.0)
	2	0	(0.0)	0	(0.0)	0	(0.0)
	3	0	(0.0)	0	(0.0)	0	(0.0)
	4	2	(9.1)	0	(0.0)	2	(6.3)
	5	8	(36.4)	6	(60.0)	14	(43.8)
	6	8	(36.4)	3	(30.0)	11	(34.4)
	7 (highest level skills)	4	(18.2)	1	(10.0)	5	(15.6)

*Being a teacher of children/adolescents.

**7-point scale.

### Prior ability to identify mental health problems (in practice)

Table 2 presents demographic characteristics of students included in hypothetical referral lists made by teachers before training (26 cases) and their respective controls. Of a total of 52 participant students, 42 (80.8%) were male. As cases and controls were matched for gender, grade, and age, differences between the two groups were not found for these three variables (p > 0.05).

**Table 2:** Demographic characteristics of cases (N = 26) and controls (N = 26)*

**Demographics**	Cases**		Controls***		p****	**Total**	
	**N**	**(%)**	**N**	**(%)**		**N**	**(%)**
Gender							
Male	21	(80.8)	21	(80.8)	1.000	42	(80.8)
Female	5	(19.2)	5	(19.2)		10	(19.2)
Age (years)							
11	1	(3.8)	1	(3.8)	0.413	2	(3.8)
12	6	(23.1)	6	(23.1)		12	(23.1)
13	6	(23.1)	10	(38.5)		16	(30.8)
14	6	(23.1)	2	(7.7)		8	(15.4)
15	5	(19.2)	6	(23.1)		11	(21.2)
16	2	(7.7)	0	(0.0)		2	(3.8)
17	0	(0.0)	1	(3.8)		1	(1.9)
School grade							
5-7	17	(65.4)	17	(65.4)	1.000	34	(65.4)
8	4	(15.4)	4	(15.4)		8	(15.4)
High school	5	(19.2)	5	(19.2)		10	(19.2)

*Cases and controls were matched by age, gender and grade.

**Cases = Students identified by teachers as possibly having mental health problems (included in hypothetical referral lists before training).

***Controls = Students not included in teachers’ hypothetical referral lists.

****Chi-square test.

Table 3 shows that cases and controls differed in relation to the presence of internalizing and externalizing problems (p = 0.003). When we compared cases and controls with both internalizing and externalizing problems (group 3) to cases and controls with no such problems (group 4), we found that teachers could identify students with a broad diversity of symptoms as being in need of mental health care, and did put them in their hypothetical referral lists (cases). No students with these mixed symptoms were found among controls (p = 0.010). When comparing cases and controls with internalizing problems only (group 1) to cases and controls with none (group 4), it was clear that teachers had a tendency to exclude students with only internalizing problems from the referral lists, showing that these problems often went unnoticed (p = 0.090). The comparison between cases and controls with externalizing problems only (group 2) with group 4 (none) did not reach statistical significance (p = 0.315), but the numbers for group 2 were small and did not allow for a definitive conclusion.

**Table 3:** Internalizing and externalizing problems in cases and controls according to the Youth Self-Report/YSR

**Internalizing and externalizing problems**	Cases*		Controls**		
	**N = 26**		**N = 26**		
	**N**	**(%)**	**N**	**(%)**	p***
(1) Internalizing only	4	(15.4)	12	(46.2)	0.003
(2) Externalizing only	1	(3.8)	3	(11.5)	
(3) Both	9	(34.6)	0	(0.0)	
(4) None	12	(46.2)	11	(42.3)	
(1) vs. (4)					0.090
(2) vs. (4)					0.315
(3) vs. (4)					0.010

*Cases = Students identified by teachers as possibly having mental health problems (included in hypothetical referral lists before training).

**Controls = Students not included in teachers’ hypothetical referral lists, matched by age, gender and grade.

***Chi-square test.

### Prior ability to identify and refer children with mental health problems (in theory)

Table 4 shows teachers’ rates of correct answers for the identification and appropriate referral of adolescents described in the vignettes before and after training. In terms of total correct answers before training, at least 76.7% of teachers could identify all five psychopathological conditions as a mental health problem and could make the appropriate referral. The rate of correct answers was greater for the vignette of conduct disorder (96.7%) and lower for the vignettes of hyperactivity (76.7%) and high risk for psychosis (76.7%). Before training, the lowest rate of correct answers within the set of six vignettes was related to normal adolescence (66.7%).

**Table 4:** Correct answers regarding identification/referral of adolescents described in vignettes before and after training (N = 30*)

**Teachers’ correct answers**		**Psychopathological conditions**					**Normal adolescence**
		**Conduct**	**Mania**	**Depression**	**Hyperactivity**	High risk**	
		**N (%)**	**N (%)**	**N (%)**	**N (%)**	**N (%)**	**N (%)**
Before training							
**Psychopathological conditions**							
	Identifies as a problem and refers to:						
	Mental health care	20 (66.7)	15 (50.0)	24 (80.0)	11 (36.7)	19 (63.3)	
	Mental health care & after-school help	9 (30.0)	9 (30.0)	0 (0.0)	12 (40.0)	3 (10.0)	
	Mental health care & other	0 (0.0)	1 (3.3)	0 (0.0)	0 (0.0)	1 (3.3)	
	Total of correct answers	29 (96.7)	25 (83.3)	24 (80.0)	23 (76.7)	23 (76.7)	
**Normal adolescence**							
	Does not identify as a problem and:						
	Does not refer						19 (63.3)
	Talk to parents						1 (3.3)
	Total of correct answers						20 (66.7)
After training							
**Psychopathological conditions**							
	Identifies as a problem and refers to:						
	Mental health care	22 (73.3)	18 (60.0)	22 (73.3)	14 (46.7)	22 (73.3)	
	Mental health care & after-school help	6 (20.0)	7 (23.3)	3 (10.0)	7 (23.3)	2 (6.7)	
	Mental health care & other	0 (0.0)	0 (0.0)	0 (0.0)	1 (3.3)	0 (0.0)	
	Total of correct answers	28 (93.3)	25 (83.3)	25 (83.3)	22 (73.3)	24 (80.0)	
**Normal adolescence**							
	Does not identify as a problem and:						
	Does not refer						24 (80.0)
	Talk to parents						0 (0.0)
	Total of correct answers						24 (80.0)

*Two teachers excluded due to incomplete data in questionnaires applied before and/or after training.

**High risk for psychosis.

### Effectiveness of the psychoeducational strategy for teachers

When we compared the rates of correct responses obtained before and after training for each vignette (Table 4), little variation was noted for the five psychopathological conditions, but there was an increase from 66.7% to 80.0% for normal adolescence. However, the teachers who made the correct identification and referral after training were not often the same as those who gave the correct answers before training; we therefore expanded our analysis. Table 5 describes the proportion of teachers who already knew how to identify and correctly refer hypothetical cases before training and continued to do so after training, as well as the proportion of teachers who were successful only after training. Table 5 shows that 90.0% of teachers already knew how to identify conduct disorder as problematic and to make the appropriate referral before training, and continued to do so after training. The rate of correct answers for conduct disorder was much higher than the rates observed for the other psychopathological conditions, especially hyperactivity (60.0%) and high risk for psychosis (62.7%). The rate of correct answers was also low for the vignette of normal adolescence (60.0%).

**Table 5:** Correct answers* regarding the identification/referral of adolescents described in vignettes and training effectiveness (N = 30**)

**Vignettes**	**Already knew how to identify and correctly refer and continued to do so**^**a**^		**Training effectiveness**^**b**^	
	**N**	**(%)**	**N**	**(%)**
**Psychopathological conditions**				
Conduct	27/30	(90.0)	1/1	(100.0)
Mania	22/30	(73.3)	3/5	(60.0)
Depression	22/30	(73.3)	3/6	(50.0)
Hyperactivity	18/30	(60.0)	4/7	(57.1)
High risk for psychosis	20/30	(62.7)	4/7	(57.1)
**Normal adolescence**	18/30	(60.0)	6/10	(60.0)

*Teachers’ correct answers before and after training.

**Two teachers excluded due to incomplete data in questionnaires applied before and/or after training.

^a^Correct answers before and after training.

^b^Correct answers after training among teachers with incorrect answers before training.

The training seemed only partially useful in helping teachers who before training could not identify and/or refer the cases that were described in the five vignettes related to psychopathological conditions. At least 50.0% of these teachers learned to identify the vignettes problematic and to make the appropriate referral. The psychoeducational strategy was also helpful to 60.0% of teachers who before training could not identify normal adolescence as an absence of problems and who could not recognize that in this case referral to a mental health service was not needed.

When we asked teachers (N = 32) at the end of training, how many students from their referral lists they had in fact referred to mental health services, only 17 answered, and no referrals had been made.

The vast majority of teachers we trained positively evaluated the quality of the psychoeducational strategy applied (93.5%). In addition, they reported that they had acquired knowledge on the subject (74.2%) and would recommend the training to teachers from other schools (96.8%). They also made suggestions about improving the quality of training by extending its duration and being more interactive by including discussion of real cases. Furthermore, teachers suggested that a continuous education program should be developed that not only encompassed the discussion of real cases, but also gave practical guidance on how to deal with disordered students in the classroom.

## Discussion

The current study may be considered pioneering in Brazil because few studies by Brazilian researchers have examined adolescent mental health in the school environment. Furthermore, evaluating the effectiveness of any interventions developed for teachers in this area has also been neglected. When observing the characteristics of teachers that participated in the study, we noted that 93.8% evaluated themselves highly in relation to their vocational skill level for teaching students older than 11 years of age. Their self-reported skill scores ranged from five to seven, with the lowest score for vocational skill being one and the highest being seven. Therefore, it is reasonable to hypothesize that teachers with greater experience in teaching adolescents and their higher-level vocational skills could have favored the effectiveness of the psychoeducational strategy proposed. Then again, more than half of the participants (53.1%) said they would change their profession if they could. However, this dissatisfaction does not seem to be associated with experience and vocational skills, but to other factors, such as underpayment, violence in schools and possibly chronic stress caused by their inability to deal with mentally disordered students in the classroom. This hypothesis concurs with that of Rothi et al. [14] who argued that teachers are professionally dissatisfied when feeling overloaded by their students’ unmet needs concerning mental health assistance.

Based on prior publications [15-17], Rothi, Leavy and Best [18] emphasize that children and adolescents with emotional and/or behavioral problems have their learning ability impaired and that they also interfere negatively with the school environment, making it unsuitable for their peers’ learning processes, both socially and academically. Therefore, whenever the teacher has difficulties managing a student’s emotional/behavioral problem, the group dynamics become so turbulent that the whole class’ learning process becomes disadvantaged.

### Prior ability to identify mental health problems (in practice)

It is interesting to note that overall the prevalence of mental health problems identified by the YSR among the controls (57.7%) was about the same as in the teacher-nominated group (53.8%). This can be explained by the teachers’ difficulties with interpreting the non-psychopathological adolescent behaviors as normal, as well as interpreting the groups of symptoms that characterize pure internalizing and pure externalizing adolescent behaviors as abnormal.

According to the YSR, all students who had mixed symptoms, comorbidity of internalizing and externalizing problems, belonged to teachers’ referral lists (cases) and students who had internalizing problems only were more frequently found among controls than among cases. Only a small number of cases and controls were identified as having externalizing problems alone. Therefore, the study’s data in this area is not precise. It is reasonable to hypothesize that more symptomatic students are more easily noticed by teachers; however, one must consider that what the teachers noticed in this mixed group was the externalizing component and we cannot be sure if they were able to tell this group apart from those with pure externalizing symptoms. Also, students who present with anxiety and/or depressive symptoms alone (internalizing problems) are rarely identified as cases because they do not disturb the classroom dynamics. Many of the students who presented with internalizing problems alone were not identified as presenting any mental health problem, probably because their behavior was mistaken for that expected in normal adolescence, such as some degree of social isolation, grumpiness or laziness. Therefore, it becomes clear that in practice, teachers have difficulty in distinguishing the normal adolescent behavior profile from those symptoms that are indicative of mental health problems, such as depression or anxiety.

It is also important to consider the interference of opinions that the teacher may have about his/her role as an educator. According to Rothi, Leavy and Best [18] teachers use certain indicators to identify students with mental health problems. For example, externalizing symptoms that are disruptive to the learning environment; changes in the usual behavior of the adolescent or the presence of abnormal behaviors when compared with their peers; difficulty in establishing and maintaining healthy relationships; and a lack of progress in learning. The authors warn the scientific community about the risk of teachers only identifying mental health problems in students with a concomitant drop in academic performance. They argue that by intervening only when situations impair learning, maintains their narrower view of their role as educators, instead of realizing their broader role, which is in the global development of students.

### Prior ability to identify and refer students with mental health problems (in theory)

Before training, the majority of teachers could recognize as problems the five psychopathological conditions described in the vignettes and appropriately refer them. This prior knowledge was also mentioned by Moor et al. [19], who showed that 52% of middle school teachers, even before undergoing training, would be capable of identifying depression in students with a confirmed diagnosis of clinical depression.

Before training, the lowest rate of correct answers for all the vignettes (66.7%) was related to normal adolescence. Therefore, teachers had difficulty distinguishing normal adolescence from adolescence with mental health problems. The consequences of this difficulty may be the lack of identification and referral of students who need clinical evaluation, as well as inappropriate referral of healthy students to mental health services. This is the reason why Couto et al. [6] emphasized the importance of deconstructing the demand for mental health services. Couto et al. observed the high frequency of students with learning difficulties or indiscipline being referred to mental health services because of their teacher’s belief that they had mental health problems. Furthermore, the negative consequences of failing to identify conduct disorders may result in students being punished for indiscipline, instead of being appropriately referred for mental health evaluation and treatment. Atkins et al. [20], cited 2002 data from the Department of Education of the United States [21], and showed a desolating scenario, in which 47% of all children and adolescents in elementary and middle school, who were classified as mentally disturbed, had been suspended or expelled from school at some point during their school career. It is necessary that teachers not only correctly identify the presence of possible mental health problems, but also properly manage problematic students in the classroom so that children do not get into a situation of negative exposure or helplessness. Therefore, despite the teachers’ prior knowledge, a psychoeducational strategy that can build their capability to distinguish normal adolescent behaviors from signs that indicate emotional and behavioral problems is still necessary to increase the rate of appropriate referral for students who need mental health care.

### Effectiveness of the psychoeducational strategy for teachers

Study results showed that 90.0% of teachers already knew how to identify the vignette of conduct disorder as a problem and to make the appropriate referral before training, and continued to know it afterwards. This rate of correct answers was much higher than the rates obtained for the other four vignettes that described psychopathological conditions, especially hyperactivity (60.0%) and high risk for psychosis (62.7%). These results suggest that because of its disruptive nature, conduct disorder symptoms easily irritate teachers and create disturbance in the classroom. Symptoms such as challenging and confronting the teacher, as well as aggressive and often violent behaviors, create an unbearable situation that favors the teachers identifying these students as problematic and referring them to specialized services. Although this hypothesis could explain our results regarding the teachers’ interpretation of vignettes, which demonstrated their theoretical knowledge, we found that they did not actually refer any of their students to mental health care.

Study results also showed that 80.0% of teachers already knew how to identify the vignette of depression as a problem and to make the appropriate referral before training. However, the detection rate of internalizing symptoms in the case-control study was very low. This fact illustrates a discrepancy between the amount of theoretical knowledge teachers have and their attitudes in practice. In a study by Pearcy, Clopton and Pope [22] sixty-four third, fourth, and fifth-grade teachers were asked to read vignettes that described boys and girls with externalizing and internalizing disorders, and to report on their prior experience in referring their own students for treatment according to different student behavior profiles. Teachers reported having referred more children with externalizing problems than with internalizing problems, even though in the vignettes they rated externalizing and internalizing problems as similarly needing referral. The discrepancy existing between teachers’ responses to hypothetical children and to their own students may be explained by the realities of the classroom environment. When analyzing vignettes, teachers have no personal involvement with the child, but when dealing with actual students, the degree of classroom disruption further influences their decision to refer the child for mental health evaluation.

Rothi, Leavy and Best [18] cited observations made by Atkinson and Hornby [23], who stated that teachers reported feeling unprepared and uncomfortable in dealing with their students’ mental health problems and that they lacked knowledge on the subject. To achieve appropriate referral, it would be beneficial for teachers to have mental health professionals training and guiding them because teachers should not be expected to assume this responsibility alone, with no support, no training, and no guidance [24].

Training was partially effective since at least 50.0% of teachers learned to identify hypothetical cases as problematic and to make the appropriate referral, and 60.0% of teachers who before training could not identify normal adolescent behavior learned to do so.

It is fundamental for teachers to know more about the psychological development of children and adolescents because they have daily contact with students in this age group. They are also in an ideal position to observe the early signs of mental health disorders. Bostock, Kitt and Kitt [25] recommended that mental health training should be included in the post-graduate curriculum for teachers so that those with less experience could be properly trained to deal with students’ mental health problems, which they will inevitably encounter during the course of their professional career.

Based on the qualitative evaluation of the training, it is reasonable to state that the psychoeducational strategy used in this study could be improved through the following initiatives: longer duration; discussion of real cases that teachers bring to training sessions, which focus not only on increasing theoretical knowledge, but also on change of attitudes; presentation of vignettes be given to each teacher in handouts instead of a slide presentation only; content improvement of the vignettes to make them longer and more detailed, which would allow a more complete characterization of psychopathological conditions noticed within the school environment; and distribution of booklets that contain the most relevant information regarding the psychoeducational strategy content, to allow future consultation and further consideration.

### Study limitations

Despite the indication that the psychoeducational strategy used was partially effective, we recognize that the study has some limitations. The sample size of teachers was small and because the study was based on a representative sample of teachers from only one public school in the center-south region of the city, it is impossible to generalize research results to all public teachers of São Paulo. Another limitation was the reduced power within the case-control study to detect any differences in the types of mental health problems observed among female students because 80.8% of the sample was composed of males. In addition, vignettes did not include anxiety, a prevalent child/adolescent disorder in the general Brazilian population (5.2%) [26]. However, this research contributed as an exploratory study to evaluate teachers’ current knowledge regarding the identification and appropriate referral of students with possible mental health problems, in theory and in practice. It also served to identify the teachers’ weakest areas in terms of their knowledge and their need for guidance from mental health specialists. This information will help us to further develop and focus future mental health training programs for public school teachers.

## Conclusions

In practice, teachers showed prior ability in identifying those students who were most symptomatic and in need of mental health assistance, but had difficulty in identifying students with internalizing problems only. In theory, the majority of teachers could already identify the vignettes that described the five psychopathological conditions as problematic and make the appropriate referral, but showed difficulty in distinguishing between normal and abnormal adolescent behaviors. The training helped increase knowledge among teachers who before training could not identify and/or refer the cases that were described in the five vignettes related to psychopathological conditions, and teachers who before training could not identify normal adolescent behaviors. The effectiveness of the psychoeducational strategy used would be improved by taking into account the suggestions that teachers made during the qualitative evaluation of the training.

These findings provide scientific evidence of the need for training Brazilian public school teachers to recognize students with possible mental health problems and to be able to differentiate them from students with behaviors considered normal in adolescence. This would allow for the early identification and treatment of those students in need of mental health care. It would also reduce the referral rate of students who exhibit normal behaviors to specialized services, which are already overloaded.

### Recommendations

To avoid failure of mental health training programs, it is necessary to use the expression “mental health problems” carefully, which may have negative connotations among educators because of the stigma usually associated with severe mental illness. We also need to recognize that teachers may be reluctant to incorporate psychiatric concepts into their training because they may assume they are being forced to diagnose students with mental health issues, a task they do not consider as part of their role. Therefore, use of more accepted terms such as “emotional and/or behavioral problems” may avoid misunderstandings and stigma. It is also essential to respect that a teacher’s principal role is as an educator and we should not try to turn them into health professionals. As educators, teachers are focused on the students’ progress and can therefore accept a partnership with health professionals when it comes to having a caring and positive attitude that will benefit the students’ global development.

Before developing a new model for mental health training, it would be useful to check for existing models and evaluate them for their effectiveness in the original sites, for any adaptations that may be needed in a new environment, and their feasibility and affordability in these new contexts. We would also recommend that prior to commencing new interventions, researchers conduct a qualitative study to become familiar with new contexts and the intended target audience, which will identify their main needs regarding central concepts to be discussed and positive attitudes to be acquired. Qualitative data obtained will guide decision-making regarding the content and procedures that should be adopted by the psychoeducational strategy. Consecutively, it is advisable to perform an exploratory quantitative pilot study to evaluate the effectiveness of the new strategy among specific audiences. Pilot study results will guide the appropriate adjustments in the training model before applying it on a larger scale. The improved model should have its effectiveness evaluated again, through a further pilot study, until it is considered to be a final model, with no further adjustments needed. This final model can then be used broadly and even be incorporated into public policies.

To guarantee an effective and sustainable mental health training program, it is necessary to have the cooperation of both school teachers and administrators to guarantee implementation and sustainability. It is also necessary to have broad lines of communication between the health and education sectors, to maintain an open dialogue between educators and mental health professionals, which ensures appropriate referrals and avoids an increase of false-positive referral rates. Mental health needs to be included in the public policy agenda for education, which would ensure continued financial support for effective training programs. Furthermore, including child development and general mental health topics in the teacher-training curriculum would familiarize these professionals with basic concepts at the beginning of their career. These topics should also be part of a teacher’s continuing education, which would enable them to develop healthy habits in the classroom and help them to deal with students who do have mental health problems.

## Competing interest

The authors declare that they have no competing interests.

## Authors’ contributions

All authors have made substantial intellectual contributions to the current article. All authors were involved in study conception and design, analysis and interpretation of data, revising the final draft critically for important intellectual content and have given final approval for the revised version to be published. AGA, TSM and RAB were also involved in acquisition of funding. MAV, AGA, TSM and RAB were also involved in field work. MAV and IAB were also involved in drafting the manuscript. In addition, the current article derives from the master thesis of the post-graduate student MAV at the Department of Psychiatry, Universidade Federal de São Paulo, Brazil, concluded in 2012, under supervision of two professors: IAB (child and adolescent psychiatrist and epidemiologist) and RAB (schizophrenia specialist and neuroscience researcher).

## Pre-publication history

The pre-publication history for this paper can be accessed here:

http://www.biomedcentral.com/1471-2458/14/210/prepub
